# Unsupervised corneal contour extraction algorithm with shared model for dynamic deformation videos: improving accuracy and noise resistance

**DOI:** 10.1186/s12938-023-01188-7

**Published:** 2024-01-08

**Authors:** Zuoping Tan, Xuan Chen, Qiang Xu, Can Yang, Xiaomin Lin, Yan Huo, Mohammad Alzogool, Riwei Wang, Yan Wang

**Affiliations:** 1 Wenzhou University of Technology, Wenzhou Economic and Technological Development Zone, Longwan District, No. 337, Jinhai Third Road, Wenzhou, 325000 Zhejiang China; 2 School of Medicine, Nankai University, Tianjin, China; 3 Clinical College of Ophthalmology, Tianjin Medical University, No. 4 Gansu Road, Heping District, Tianjin, 300020 China; 4 Tianjin Key Laboratory of Ophthalmology and Visual Science, Tianjin Eye Hospital, Tianjin Eye Institute, Nankai University Affiliated Eye Hospital, Tianjin, China; 5 Nankai Eye Institute, Nankai University, Tianjin, China

**Keywords:** Unsupervised, Corneal contour extraction, Shared model

## Abstract

**Background:**

In this study, an automatic corneal contour extraction algorithm with a shared model is developed to extract contours from dynamic corneal videos containing noise, which improves the accuracy of corneal biomechanical evaluation and clinical diagnoses. The algorithm does not require manual labeling and completes the unsupervised semantic segmentation of each frame in corneal dynamic deformation videos based on a fully convolutional deep-learning network using corneal geometry and texture information.

**Results:**

We included 1027 corneal videos at Tianjin Eye Hospital (Nankai University Affiliated Eye Hospital) from May 2020 to November 2021. The videos were obtained by the ultra-high-speed Scheimpflug camera, and then we used the shared model mechanism to accelerate the segmentation of corneal regions in videos, effectively resist noise, determine corneal regions based on shape factors, and finally achieve automatic and accurate extraction of corneal region contours. The Intersection over Union (IoU) of the extracted and real corneal contours using this algorithm reached 95%, and the average overlap error was 0.05, implying that the extracted corneal contour overlapped almost completely with the real contour.

**Conclusions:**

Compared to other algorithms, the method introduced in this study does not require manual annotation of corneal contour data in advance and can still extract accurate corneal contours from noisy corneal videos with good repeatability.

## Background

The cornea is the transparent part in front of the outermost layer of the eyeball wall, which constitutes two-thirds of the refractive power. It is mainly composed of nonvascular connective tissue and has the mechanical characteristics of a biological tissue. The corneal biomechanical properties have been proven to play an essential role in maintaining its structure, diagnosing ectatic diseases, screening glaucoma, and evaluating corneal refractive surgery [1, 2]. Therefore, the study of corneal biomechanics has gained popularity [3]. The Corneal Visualization Scheimpflug Technology (Corvis ST; Oculus, Wetzlar, Germany) is an existing in vivo corneal biomechanical evaluation device. It flattens the cornea twice and automatically captures the entire corneal deformation process after an external air puff at a speed of 4330 fps using an ultra-high-speed Scheimpflug camera, which is the most primitive and direct reflection of corneal biomechanics [4, 5]. However, corneal biomechanics, measured in dynamic states, still represent a new area of interdisciplinary research. The integration of ultra-high-speed Scheimpflug imaging with the proposed algorithm has enormous potential as a research and clinical tool to accurately evaluate in vivo biomechanical properties of the cornea. Therefore, accurate extraction of corneal contours from dynamic videos is the premise of accurate calculation and evaluation of corneal biomechanical characteristics and an important basis for further establishment of the corneal biomechanical model.

Most existing corneal contour extraction methods are based on traditional edge detection methods [6–10]. Magdalena et al. proposed a corneal outer edge detection method based on Corvis ST with a minimum average error of 0.16% [6]. Kasprzak et al. performed repeated Gaussian smoothing on the detected outer edge of the original cornea [7]. However, edge detection is not precise enough to locate the corneal edge and often leads to the partial loss of the corneal contour.

The semantic segmentation framework of a deep convolutional neural network has strong feature extraction ability, and its classical model and many improved models are widely used in image semantic segmentation tasks [11–14]. Two convolutional network models, SqueezeNet [15] and GoogLeNet [16], are applied to the segmentation of tumor images, which improves efficiency and achieves high accuracy [13]. In addition, the latest transformer framework has been applied to image segmentation tasks; however, the framework must be trained with several data sets before excellent results can be obtained. For medium-sized data sets (such as ImageNet-21 k [17]), the performance of the transformer model is not as good as that of the convolution architecture [18]. Owing to the small size of the corneal deformation video data set, this study proposes an unsupervised automatic corneal contour extraction algorithm based on a shared model using a convolution computing architecture.

The algorithm is based on a fully convolutional deep-learning network without the tedious and time-consuming manual marking of the corneal contour image for training. Using the powerful ability of the FCN to represent and combine the image details [19], it extracts the low- and high-order features of the image, assigns the same label to the corneal region pixels in each frame of the corneal deformation videos, and realizes the unsupervised semantic segmentation of the corneal image region. The model parameter-sharing mechanism is designed to accelerate the segmentation of each frame image in the video, effectively improving the anti-noise ability and computational efficiency. The corneal region is screened according to the shape factor and finally realizes the high-precision automatic extraction of the noise-robust unsupervised corneal contour.

## Results

The method proposed is an unsupervised corneal contour extraction for a single frame video, which do not require training data for weight parameters, and its training stage is the inference process. In total, 1027 videos were analyzed for evaluating the performance. The image labeled actual corneal contours manually are served as the ground truth.

Intersection over Union (IoU) is a measure of the accuracy of the detected object [20]. It calculates the overlap ratio between the output image and the ground truth; in the words, if there are more intersections between the output and the ground truth, it indicates that the accuracy of the model is higher. Therefore, it is the most popular evaluation metric used in semantic segmentation tasks in the field of deep learning [21]. In this study, the overlap error [22] was used to assess the accuracy of corneal contour extraction and is defined as follows:

$${\mathbf{E}}\, = \,1 - {\text{IoU}} = 1 - \frac{{{\text{Area}}\left( {{\text{S}} \cap {\text{G}}} \right)}}{{{\text{Area}}\left( {{\text{S}} \cup {\text{G}}} \right)}}$$

where S and G denote the extracted and actual corneal contours, respectively. From the above equation, it can be deduced that when the extracted corneal contour and true contour coincide exactly, the value of IoU is 1, and the value of **E** is the minimum value of 0. While the extracted corneal contour and the real contour have no overlapping parts, the value of IoU is 0, and the value of **E** is the maximum value of 1. That is, the smaller the **E** value is, the more accurate the extracted corneal contour.

**Fig. 1:**
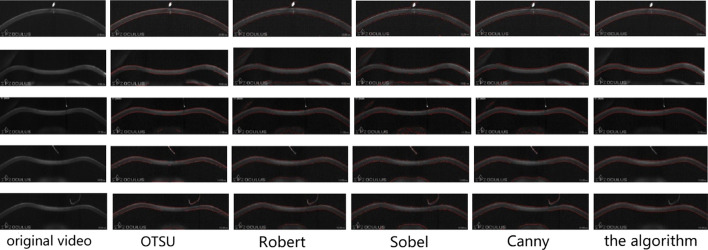
The Comparison of extraction effect for different methods

For corneal contour extraction in corneal force deformation video streams, 1027 videos are all analyzed in total. To evaluate the accuracy, we choose five videos with much noise to demonstrate the anti-noise performance, and the effect of the proposed algorithm was compared with that of existing algorithms, such as OTSU, Robert, Sobel, Canny, as shown in Fig. 1.

From Fig. 1, it can be seen that when the video contains noise, the contours extracted by existing methods are generally disturbed by noise, and the extracted corneal contours appear discontinuous, abnormal, or even mistake the noise as pixel points on the corneal contours. However, the proposed method in this study is not only noise-resistant, but also has a high accuracy of corneal contour extraction.

To quantify the difference in accuracy between the proposed method and the existing corneal contour extraction methods, the mean and variance of the overlap error were calculated separately for the same videos using OTSU, Robert, Sobel, Canny, and the proposed method; the results are listed in Table 1.

**Table 1:** Comparison of overlap error (mean ± std)

	Video1	Video2	Video3	Video4	Video5
OTSU	0.31 ± 0.13	0.64 ± 0.02	0.50 ± 0.22	0.24 ± 0.10	0.50 ± 0.10
Robert	0.54 ± 0.05	0.72 ± 0.01	0.69 ± 0.11	0.55 ± 0.07	0.66 ± 0.04
Sobel	0.54 ± 0.07	0.71 ± 0.04	0.66 ± 0.12	0.51 ± 0.07	0.68 ± 0.04
Canny	0.30 ± 0.15	0.66 ± 0.03	0.58 ± 0.17	0.32 ± 0.13	0.56 ± 0.05
The shared model	0.05 ± 0.02	0.05 ± 0.01	0.05 ± 0.02	0.05 ± 0.01	0.05 ± 0.02

Table 1 shows that the mean value of the overlap error of the existing contour extraction methods is greater than 0.2, while the mean value of the overlap error of the proposed corneal contours extracted algorithm is only 0.05. This implies that the corneal contours extracted using the proposed method almost overlap with the real contours, even in the presence of noise interference.

To elaborate on fully convolutional network architecture, a U-Net, which was older but still more recent, was used to replace the FCN for the feature extraction module. The mean value and variance of the overlap error for Video 1, Video 2, Video 3, Video 4, and Video 5 are 0.42 ± 0.04, 0.11 ± 0.02,0.09 ± 0.02,0.18 ± 0.0, and 0.10 ± 0.02, respectively.

Furthermore, the overlap error with different activation functions, such as ReLU [23], MiSH [24], Pish [25], LeakyReLU [26], is compared, and the comparative result is shown in Table 2.

**Table 2:** Overlap error for different activation functions (mean ± std)

	Video1	Video2	Video3	Video4	Video5
ReLU	0.05 ± 0.02	0.05 ± 0.01	0.05 ± 0.02	0.05 ± 0.01	0.05 ± 0.02
MiSH	0.06 ± 0.05	0.10 ± 0.03	0.14 ± 0.07	0.13 ± 0.02	0.13 ± 0.05
Pish	0.27 ± 0.02	0.81 ± 0.16	0.83 ± 0.06	0.80 ± 0.01	0.82 ± 0.01
LeakyReLU	0.07 ± 0.02	0.07 ± 0.01	0.06 ± 0.02	0.03 ± 0.01	0.05 ± 0.02

Table 2 shows that the mean overlap error for ReLU activation function is smaller than other different activation function.

**Fig. 2:**
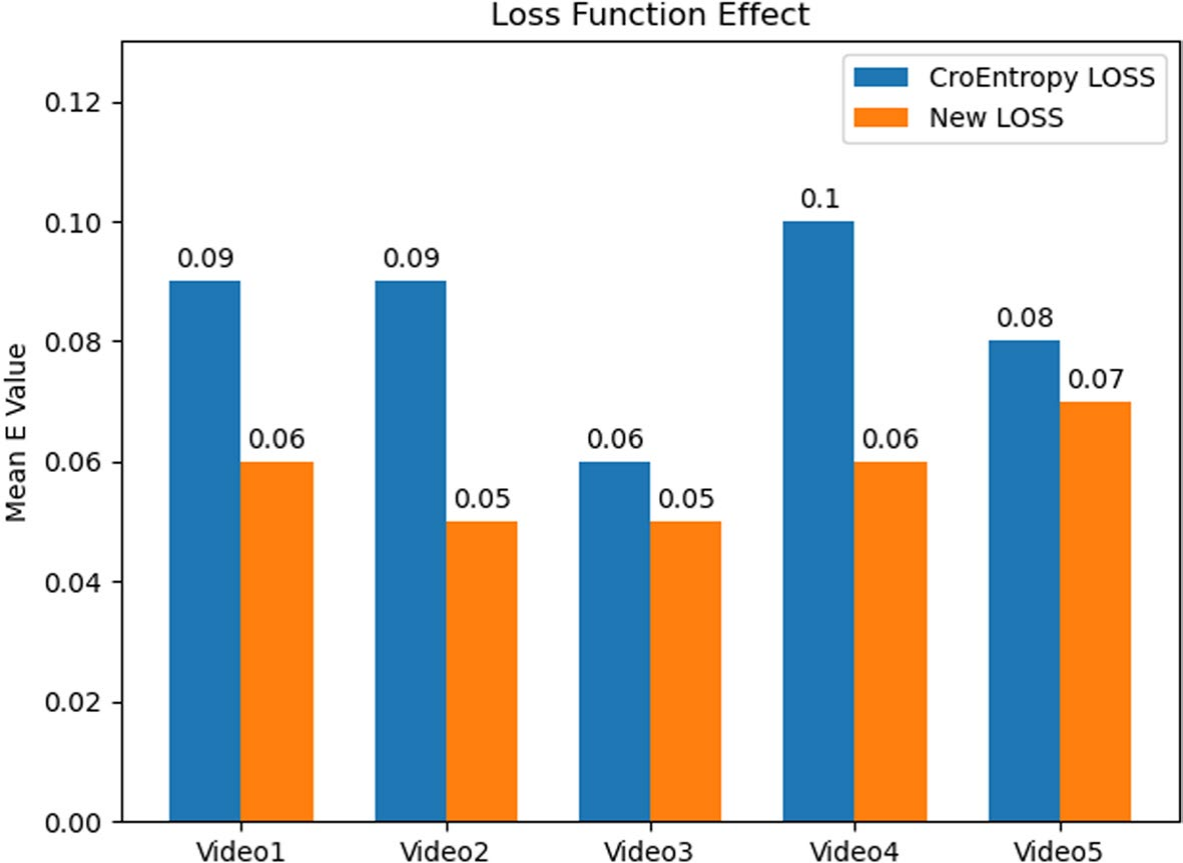
Comparison of different loss functions

For further analysis of the effect using different loss functions, the overlap error **E** was calculated for the above five videos using the cross-entropy loss function and improved loss function. The results are shown in Fig. 2.

From Fig. 2, it can be observed that the average overlap error value **E** obtained using the improved loss function is significantly smaller than the **E** value of the cross-entropy loss function. That is, the corneal contours extracted using the improved loss-function model were closer to the real corneal contours.

The corneal area in the video was relatively fixed, and the range of corneal variation between adjacent images was small. Therefore, this study used a shared mode. For the above videos, because the unsupervised corneal contour extraction method has a certain randomness, a shared model and a randomly initialized parameter model were used for contour extraction, and both were repeated three times. The comparison results are listed in Table 3.

**Table 3:** Comparison results (mean ± std)

	Video1	Video2	Video3	Video4	Video5
Random initialized model	0.09 ± 0.03	0.99 ± 0.09	0.07 ± 0.04	0.07 ± 0.03	0.07 ± 0.03
	0.04 ± 0.02	0.44 ± 0.34	0.16 ± 0.10	0.54 ± 0.37	0.07 ± 0.04
	0.05 ± 0.02	0.13 ± 0.11	0.99 ± 0.03	0.06 ± 0.04	0.24 ± 0.23
The shared model	0.05 ± 0.03	0.06 ± 0.02	0.05 ± 0.02	0.07 ± 0.04	0.06 ± 0.02
	0.05 ± 0.02	0.05 ± 0.01	0.05 ± 0.02	0.05 ± 0.01	0.05 ± 0.02
	0.06 ± 0.08	0.06 ± 0.01	0.05 ± 0.02	0.06 ± 0.04	0.06 ± 0.02

It is evident from the table above that the average overlap error value for each video was less than 0.1 using the shared model to guide the training. Although the average overlap error using the random initialization parameter model was significantly larger than that using the shared model, the repeatability of the shared model was also much better. This confirms that the shared-parameter model can effectively resist noise interference and extract more complete and accurate corneal contours.

**Fig. 3:**
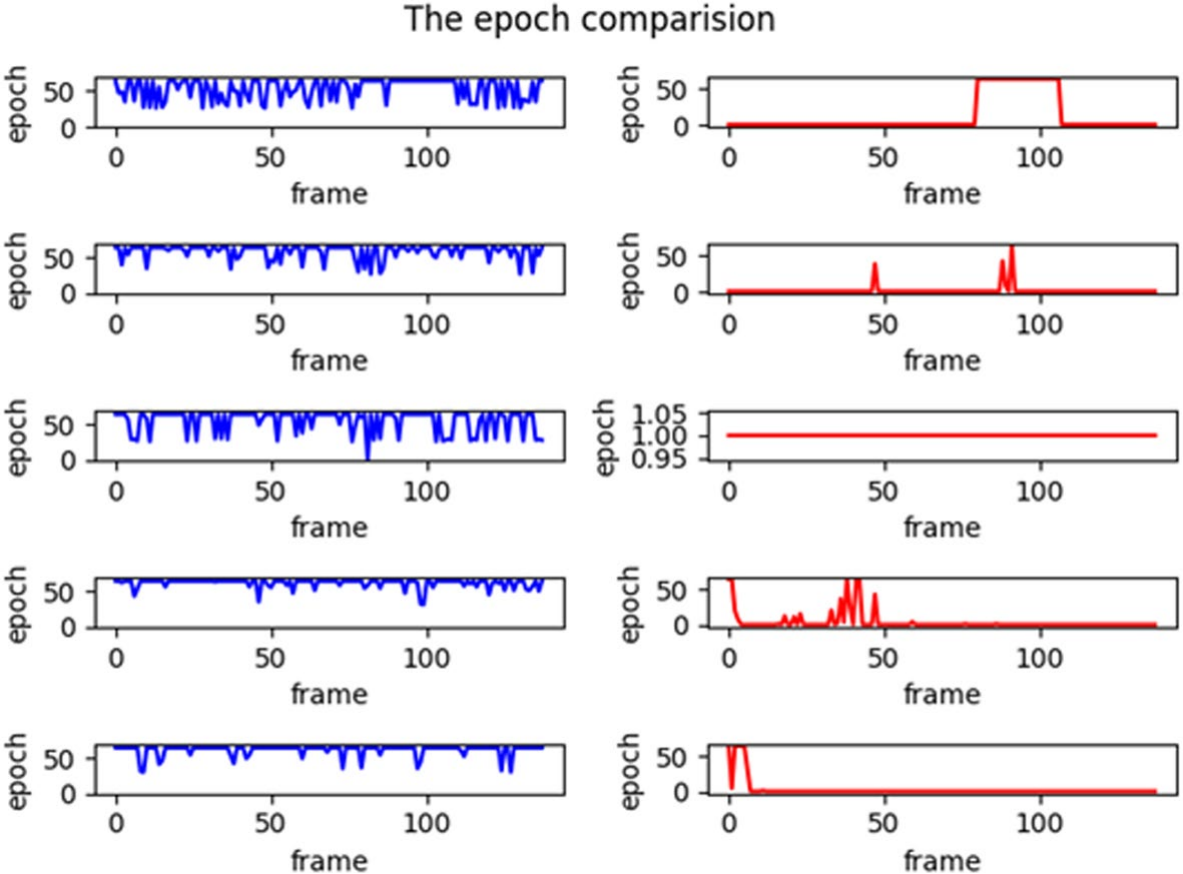
Comparison of the epochs when the loss function is 0.1

To evaluate the training efficiency, the change process of the loss function is analyzed. In other words, when the loss function is less than 0.1 at first time, the current number of iteration epoch was remembered, and the smaller value is, the higher the efficiency is. For the above video, the number of iterations of the shared model and the randomly initialized parameter model were calculated separately, and the comparison results are shown in Fig. 3.

From the above figure, it can be seen that the number of iterations using the shared model with a loss function equal to 0.1 is much smaller than that of the random initialized parameter model. Particularly, the shared model can reduce training time more effectively.

## Discussion

In this study, we described an unsupervised corneal contours extraction based on the fully convolutional network architecture. A total of 1027 videos were analyzed and a good performance was demonstrated on the extraction accuracy as well as noise resistance. We show that a fully convolutional network trained pixels-to-pixels on semantic segmentation can apply for automatic extraction of corneal contours, and the good initializing of the model parameters is crucial. Otherwise, some parts of the network may be overly activated, while others may not contribute. To our knowledge, this is the first work to apply unsupervised corneal contours extraction using a shared model mechanism.

Existing studies on corneal contours extraction were mostly supervised. Wangyi et al. proposed an automatic extraction algorithm for corneal image contours using swept-frequency optical coherence tomography [8]. The algorithm divides the image into high and low signal-to-noise ratio regions for processing. Using the peak point positioning method and combining the actual contour information of the cornea as a tradeoff factor, corneal contour positioning can be realized. The obtained corneal thickness was closer to the actual value, and the average extraction accuracy improved by 4.9%. However, this method requires a large amount of annotation data, and the annotation process is lengthy and prone to human error. Ji et al. proposed a method based on edge detection and quintic polynomial approximation [9]. This method can achieve good results when there are small amounts of noise and artifacts in the image. However, owing to the accuracy of the corneal edge detection algorithm and the limitations of the polynomial fitting method, individual differences cannot be accurately considered in the process of dynamic corneal changes. Koprowski et al. proposed a corneal contour edge detection method based on Otsu segmentation and the Canny operator [10]. This method provides new clues for extracting contours after segmenting the image and achieves better results. However, it does not consider how to extract the contour from videos or the impact of the correlation between video frames on corneal contour extraction. Jiang et al. explored a retinal vascular image segmentation method based on the deep fully convolutional network (FCN) model [14]. This method performs intensive pixel-by-pixel prediction from the perspective of full size with an average accuracy of 97%. However, all these methods only obtained satisfactory results for images with or without a small amount of noise. If the image quality is low or the noise level is high, extraction of the corneal contour cannot meet the clinical requirements.

Considering the presence of noise in corneal deformation videos and the lack of high-quality annotation data, this study introduced the perspective of contour calculation after segmenting. Unsupervised semantic segmentation was performed on each frame of the corneal dynamic video using a shared model mechanism to accelerate image segmentation and effectively resist noise. Subsequently, the corneal region is selected based on the shape factor to accurately extract the corneal contour. In addition, the shared model mechanism is proposed in this study. The effective use of known model parameters for the initialization improves the accuracy of semantic segmentation and speeds up the training process. By loading the model parameters of the previous frame, the FCN obtained almost ideal semantic segmentation of the corneal image, and after fewer iterations, a more complete and accurate corneal region could be obtained. For random noise in the corneal video, the effective model parameters of the previous frame are used to initialize the network, so that the interference of random noise data in the current frame can be effectively avoided. Therefore, the proposed method not only reduces the training time, but also has high accuracy and stability for corneal images with noise, which shows good noise immunity. The experimental results also fully validate this conclusion.

## Conclusions

In this study, we developed an unsupervised automatic corneal contour extraction algorithm based on a shared model of dynamic corneal deformation videos. Low- and high-order features were extracted from the image using a fully convolutional deep-learning network. Pixels with continuous spatial positions, similar shapes, and textural features were grouped together, and shape factors were designed to identify the corneal region. To resist random noise interference in the image, the training was guided by a shared model mechanism. Compared to the OTSU, Robert, Sobel, Canny, and other algorithms, the method introduced in this study does not require manual annotation of corneal contour data in advance and can still extract accurate corneal contours from noisy corneal videos with good repeatability. Accurate corneal contour extraction is a prerequisite for calculating biomechanical parameters, this provides a promising way for the accurate characterization of corneal biomechanics, further providing more reliable guidance for doctors to diagnose corneal ectatic diseases, screen for glaucoma, and predict corneal refractive surgery from the perspective of evaluating corneal biomechanics.

## Methods

### Data collection

The data set was retrospectively collected at Tianjin Eye Hospital (Nankai University Affiliated Eye Hospital) from May 2020 to November 2021 and analyzed in December 2022. A total of 1027 videos were captured by Corneal Visualization Scheimpflug Technology (Corvis ST), which applanates the cornea using a puff of air and the corneal deformation videos are recorded by a Scheimpflug high-speed camera only when the “Quality Control Score” displayed “OK”. The video frame rate is 13 fps. Each video had 139 total frames with a total time of 31.88 ms.

### Process

First, the Slic algorithm [27] was introduced to pre-group pixels according to the spatial continuity of pixels in the video of corneal force deformation; then, the unsupervised semantic segmentation of corneal images was completed by extracting the low and high features of the images based on corneal geometry and texture similarity information through a fully convolutional deep-learning network. For continuity of the corneal region between video frames, a shared model mechanism was designed to accelerate the segmentation of the corneal region in the video and improve the noise immunity performance. The corneal region was then filtered by the shape factor, and finally, contour extraction of the corneal region was completed. The pseudocode is presented in Algorithm 1.

**Algorithm 1** 
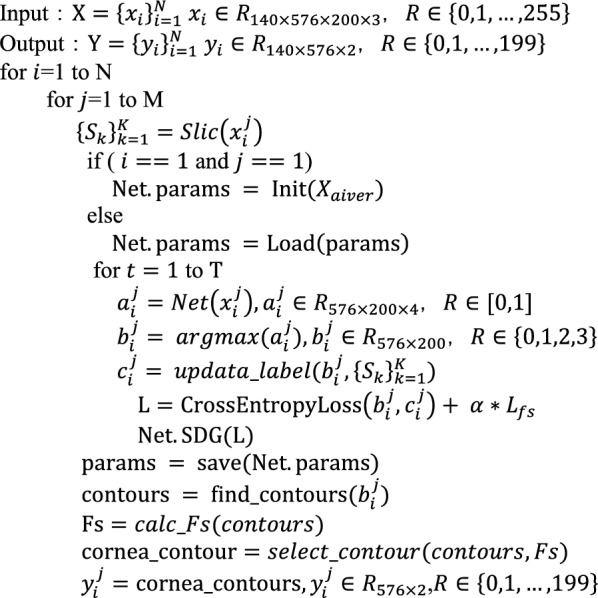


### Image semantic segmentation

FCNs have great potential for image detail feature extraction and show good performance in unsupervised image segmentation [20, 28, 29]. In this study, to achieve unsupervised semantic segmentation of corneal images, we extracted the color and texture features of each local pixel region for the Corvis ST corneal dynamic deformation video stream using an FCN, predicted the unknown clustering labels for each frame image in the video stream, learned the optimal network parameters for pixel clustering, and extracted the pixel points in each cluster label.

**Fig. 4:**
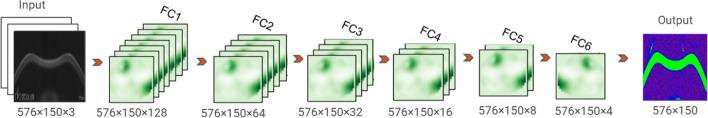
Structure of a fully convolutional network

In this study, the input is the corneal dynamic deformation videos ${\text{X}}={\left\{{x}_{i}\right\}}_{i=1}^{N}$, and we propose a distance metric $D^{\prime} = \sqrt {\left( {\frac{{d_{c} }}{{\text{m}}}} \right)^{2} + \left( {\frac{{d_{s} }}{S}} \right)^{2} }$ to pre-group images, where m = 10, $S=sqrt(N/K)$, $N$ is the number of pixels in the image ${x}_{i}^{j}$, $K$ is the number of pre-groups, ${d}_{c}$ is the color distance in Lab color space, which is defined as ${d}_{c}=\sqrt{{{{({l}_{j}-{l}_{i})}^{2}+({a}_{j}-{a}_{i})}^{2}+({b}_{j}-{b}_{i})}^{2}}$, and the spatial distance ${d}_{s}=\sqrt{{{({x}_{j}-{x}_{i})}^{2}+({y}_{j}-{y}_{i})}^{2}}$. For all pixel points of the $j$ th frame in the $i$ th video, we calculate the distance metric ${D}^{\mathrm{^{\prime}}}$. We assigned the same labels to spatially contiguous pixels with similar color or texture features, and different clustering labels to dissimilar neighboring pixels. Thus, we obtain a set of K pixel indices, ${\left\{{S}_{k}\right\}}_{k=1}^{K}$. Image ${x}_{i}^{j}$ is input to a six-layer FCN, and the structure is shown in Fig. 4.

Six convolution units are computed for image ${x}_{i}^{j}$. Each convolution unit consists of a convolution calculation, batch normalization, and a ReLU activation function. The parameters of each network layer are listed in Table 4.

**Table 4:** Parameters of fully convolutional network

Layer	Type	Maps	Size	Kernel	Stride	Padding	Activation
In	Input	3 (RGB)	576 × 150	–	–	–	–
C1	Conv	128	576 × 150	3 × 3	1	1	BatchNorm, ReLU
C2	Conv	64	576 × 150	1 × 1	1	0	BatchNorm
C3	Conv	32	576 × 150	3 × 3	1	1	BatchNorm, ReLU
C4	Conv	16	576 × 150	1 × 1	1	0	BatchNorm
C5	Conv	8	576 × 150	3 × 3	1	1	BatchNorm, ReLU
C6	Conv	4	576 × 150	1 × 1	1	0	BatchNorm
Out	–	–	576 × 150	–	–	–	Softmax

The detailed image feature mapping ${a}_{i}^{j}=\left\{W{x}_{i}^{j}+b\right\}$ was obtained using convolution networks, and the dimension with the maximum value was selected as the clustering label ${b}_{i}^{j}$ for each pixel, where ${b}_{i}^{j}= argmax({a}_{i}^{j})$; that is, each pixel was assigned to the closest point. Then, the pixel points in the grouping set ${\left\{{S}_{k}\right\}}_{k=1}^{K}$ are counted, the cluster label with the largest number in each ${S}_{k}$ is selected as the label for that grouping, and all pixel point labels ${c}_{i}^{j}$ are updated.

To achieve semantic segmentation of corneal images using a fully convolutional deep-learning network, two processes must be repeated alternately. (1) to predict the pixel labels ${c}_{i}^{j}$ using fixed network parameters, and (2) to update the network parameters $W$ and $b$ use the predicted pixel labels, and to identify all points belonging to the corneal area. The former corresponds to the forward learning of the network, and the latter corresponds to the backpropagation of the error. The cross-entropy loss function is often used for image semantic segmentation, which can compensate for the defect in which the derivative form of the sigmoid activation function is prone to saturation to a certain extent and avoids gradient dispersion in the process of gradient descent calculation.

In this study, we introduce the shape factor as an additional term for the cross-entropy loss function to address the following characteristics: the corneal region does not account for a large proportion of pixels in each frame, the corneal spatial location is relatively fixed, and the change in its geometry is small during the entire deformation process. ${\text{Fs}}$ is defined as follows:

$${\text{Fs}}=\frac{{\Vert L\Vert }^{2}}{4\pi S}$$

where *L* is the perimeter of the area enclosed by a contour, and *S* is the area enclosed by a contour.

During corneal deformation, the perimeter and contour area change simultaneously, and the shape factor remains unchanged. The original cross-entropy loss function ${L}_{ce}$ and shape factor loss function ${L}_{fs}$ are combined, and the improved loss function is defined as follows:

$$L={L}_{ce}+ \alpha \times {L}_{fs}$$

where ${L}_{ce}$ is the cross-entropy loss function; the shape factor loss function.

${{\varvec{L}}}_{{\varvec{f}}{\varvec{s}}}=\left|{F}_{s}-C\right|$; $C$ is a constant denoting the cornea-specific shape factor (here, the value is 10); ${F}_{s}$ denotes the shape factor; and $\alpha$ is a weighting factor (here, the value is equal to 0.1).

The improved loss function takes the absolute value of the difference between the shape factor of the region in the segmented image, where the corneal category label is located and the constant of the true corneal shape factor as an additional term. The closer the corneal category label in the segmented image and the true shape factor of the cornea, the smaller the shape factor loss function ${{\varvec{L}}}_{{\varvec{f}}{\varvec{s}}}$. That is, the improved loss function enhances the focus on the specific shape of the cornea in the training process of the FCN. It optimizes pixel labels and shape features simultaneously, updates network parameters using stochastic gradient optimization, and alternates between iterative labels and contour shape features. Thus, it performed well in the semantic segmentation of corneas in videos of corneal deformation.

### Corneal area detection and contour extraction

In a fully convolutional deep-learning network, neighboring pixels with similar textures, colors, luminance, and other features are clustered into irregular blocks of pixels with some visual significance. That is, pixel points with similar features are assigned the same category labels. However, extracting the corneal contours first requires determination of the type of label that represents the corneal region. Considering that the corneal region accounts for only a small percentage, it is relatively fixed in the image and the corneal always maintains its inherent shape during corneal deformation. The shape factor ${\text{Fs}}$ described in the previous section was adopted. The ratio of the perimeter to the area of each label type was calculated to filter out the corneal region, and boundary extraction of the corneal region was performed to obtain the corneal contour pixel points in each frame of the image in the video stream.

### The shared model

**Fig. 5:**
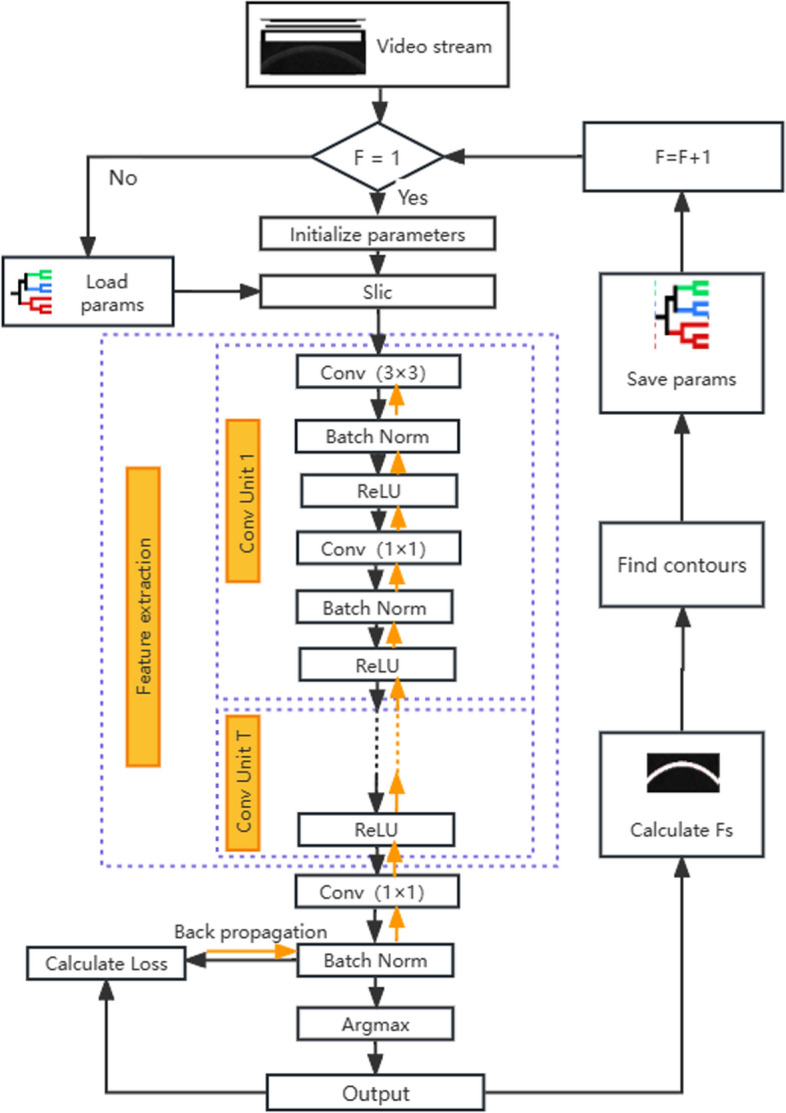
Shared model

A fully convolutional deep-learning network extracts image features through each layer of network learning, and the features learned layer-by-layer range from simple to complex. The features learned at the superficial level are simple edges, corner points, and textures, whereas those learned at the deeper level are more complex and abstract, with contour details and thicknesses. In the Corvis ST corneal dynamic deformation video, the corneal region was located in a relatively stable space with similar texture characteristics. The cornea retains its specific shape characteristics throughout the force–deformation process. The changes between adjacent frames were small and the underlying features were similar. Thus, the shallow network parameters of the FCN were similar, whereas the deep network parameters differed. Therefore, this study proposes a shared-model mechanism based on the characteristics of Corvis ST videos. That is, the model parameters of the previous frame were used to initialize the adjacent frame images. Finally, the model was thoroughly trained. This process is illustrated in Fig. 5.

## Data Availability

Data are available from the corresponding author on reasonable request.
